# Trends and Patterns of Animal Poisoning in Thailand: A 10-Year Retrospective Study from Ramathibodi Poison Center

**DOI:** 10.3390/vetsci13040325

**Published:** 2026-03-27

**Authors:** Phantakan Tansuwannarat, Satariya Trakulsrichai, Kitisak Sanprasert, Sekkarin Ploypetch, Nastayarin Ariyaviraplorn, Achara Tongpoo

**Affiliations:** 1 Chakri Naruebodindra Medical Institute, Faculty of Medicine Ramathibodi Hospital, Mahidol University, Samut Prakan 10540, Thailand; 2 Ramathibodi Poison Center, Faculty of Medicine Ramathibodi Hospital, Mahidol University, Bangkok 10400, Thailand; 3 Department of Emergency Medicine, Faculty of Medicine Ramathibodi Hospital, Mahidol University, Bangkok 10400, Thailand; 4 Department of Clinical Sciences and Public Health, Faculty of Veterinary Science, Mahidol University, 999 Phutthamonthon Sai 4 Road, Salaya, Phutthamonthon, Nakhon Pathom 73170, Thailand

**Keywords:** veterinary toxicology, pesticide toxicity, poison center surveillance, companion animals, clinical outcomes, sentinel surveillance, One Health

## Abstract

Animal poisoning remains an underreported public health and veterinary concern in many low- and middle-income countries where comprehensive surveillance systems are limited. This study was initiated to describe the epidemiology, clinical characteristics, and outcomes of animal poisoning cases reported to a national poison center in Thailand over a 10-year period. We performed a retrospective review of cases recorded in the Ramathibodi Poison Center Toxic Exposure Surveillance System between 2015 and 2024. Descriptive statistics were used to summarize species distribution, exposure categories, clinical signs, treatment, and outcomes, and comparisons were conducted to identify factors associated with mortality. A total of 118 poisoning cases were identified, with annual numbers increasing over time. Companion animals accounted for most exposures (93.2%), particularly dogs. Pesticides were the most common toxic agents, followed by household products, pharmaceuticals, and plant toxins. Neurological signs were the predominant clinical presentation. Respiratory compromise and neurological involvement at presentation were significantly associated with mortality. Overall survival was 88.1%. Fatalities were mainly linked to exposure to highly toxic pesticides or plants, including confirmed cassava-associated cyanide poisoning in elephants. This study highlights preventable environmental toxic risks affecting animals in Thailand and demonstrates the value of centralized poison surveillance. Strengthening pesticide safety practices and integrating veterinary toxicology into broader public health monitoring may reduce avoidable poisonings within shared human–animal environments.

## Simple Summary

Animal poisoning remains an overlooked but preventable threat to animal health in many parts of the world. This study examines ten years of poisoning cases reported to a poison consultation center in Thailand, providing a rare long-term view of real-world exposures. Companion animals, particularly dogs, accounted for most cases, and pesticides were the leading toxic agents. Animals arriving with breathing or neurological problems faced a markedly higher risk of death. The dataset also captured unusual but important events, including cyanide poisoning in elephants following cassava ingestion, highlighting region-specific hazards that may escape conventional reporting systems. Although these findings represent consultation-based surveillance rather than population incidence, they reveal actionable patterns of risk within shared human–animal environments. Strengthening pesticide safety practices, improving early clinical recognition of severe poisoning, and expanding poison consultation networks could reduce avoidable fatalities. The study underscores how veterinary toxicology can serve as an early warning system for environmental threats within a One Health context.

## 1. Introduction

Animal poisoning is a persistent yet underreported component of the global toxicological burden. While human poison surveillance systems are well established in many countries, comprehensive monitoring of animal exposures remains uneven, particularly in low- and middle-income regions. Companion animals often live together in the home with humans and are frequently exposed to the same hazardous substances, positioning them as both victims of preventable toxic events and sentinels of environmental risk. Reports from Europe and North America consistently show that household chemicals, pesticides, pharmaceuticals, and toxic plants account for the majority of veterinary poison consultations [1,2,3]. However, comparable long-term surveillance data from Southeast Asia remain limited.

The toxicological landscape of animal exposure reflects changing patterns of human relationships with animals, agricultural practices, and product availability. Pesticides remain a dominant cause of poisoning worldwide despite advances in regulation and formulation [4]. Organophosphates, carbamates, bipyridyl herbicides such as paraquat, pyrethroids, and commonly used rodenticides, including anticoagulant compounds such as warfarin and brodifacoum as well as metal phosphides such as zinc phosphide, remain important causes of severe poisoning in animals, often mirroring toxicological patterns observed in humans [5,6,7]. Similarly, unintended exposure to human medications is an increasingly recognized source of veterinary emergency, driven by expanding access to pharmaceuticals within households [8]. The overlap between animal and human toxic risks underscores the importance of surveillance frameworks that integrate veterinary toxicology into a broader One Health (human, animal, and environmental health) perspective.

Clinical signs of poisoning in animals are often non-specific, complicating early recognition. Neurological depression, seizures, hypersalivation, and respiratory distress are frequently reported across multiple toxicant classes [9,10]. The outcome is strongly influenced by time to intervention, toxicant dose, and species-specific susceptibility. Centralized poison center databases have proven invaluable in capturing these patterns in real time, allowing the identification of emerging hazards and informing preventive strategies. Such registries function not only as clinical resources but also as epidemiological observatories.

In Thailand, like in many other rapidly developing countries, agricultural chemicals are widely used, while pet ownership is also increasing. Despite the prevalence of these risk factors, systematic national data on animal poisoning remain scarce. Previous Thai studies have largely focused on single clinics or short observation periods [11], limiting the ability to detect longitudinal patterns. The Ramathibodi Poison Center operates as a national consultation hub and maintains a validated surveillance system reviewed by clinical toxicologists, providing a unique opportunity to examine long-term exposure trends.

The present study analyzed 10 years of animal poisoning reports captured by a centralized poison surveillance system. By characterizing species distribution, exposure categories, clinical presentation, management, and outcomes, this work aimed to provide a poison center–based epidemiological overview. Although the dataset reflects consultation records rather than population incidence, it functions as a sentinel surveillance source capable of identifying meaningful exposure trends and high-risk toxic agents in real-world settings. Beyond its relevance to veterinary medicine, these findings contribute to environmental toxicology surveillance and highlight preventable risks embedded within shared human–animal ecosystems.

## 2. Materials and Methods

### 2.1. Study Population

This retrospective study was conducted at the Ramathibodi Poison Center, a division of Ramathibodi Hospital, a tertiary university hospital in Thailand that provides nationwide, 24-h telephone consultation for veterinary professionals and the general public. Data from January 2015 to December 2024 were extracted using the Ramathibodi Poison Center Toxic Exposure Surveillance System (RPCTESS). To accurately reflect the center’s real-world utilization, a “poisoning case” was defined broadly to include any recorded inquiry regarding an actual or suspected toxic exposure. This encompassed exposures without subsequent clinical signs and suspected exposures lacking definitive toxicant confirmation. All cases coded as “Animal poisoning” or “Pet poisoning” were included, all of which underwent daily verification by senior poison information specialists and clinical toxicologists. Cases with incomplete data that precluded the analysis of primary and secondary outcomes were excluded. The study was approved by the Institutional Review Board of the Faculty of Medicine Ramathibodi Hospital, Mahidol University (COA. No. MURA2025/495), with all patient and owner identifiers removed prior to analysis.

### 2.2. Statistical Analysis

Descriptive statistics were first calculated to summarize the study variables. Continuous variables are presented as mean ± standard deviation when normally distributed and as median with interquartile range (IQR) when the distribution departs from normality. Categorical variables are expressed as counts and percentages. Comparisons between groups were performed using Student’s t-test for normally distributed continuous variables and the Mann–Whitney U test for non-normally distributed data. Categorical data were analyzed using either a chi-square test or Fisher’s exact test. A *p*-value < 0.05 was considered statistically significant. Statistical analyses were carried out using Stata version 16.1 (StataCorp, College Station, TX, USA).

## 3. Results

**Figure 1:**
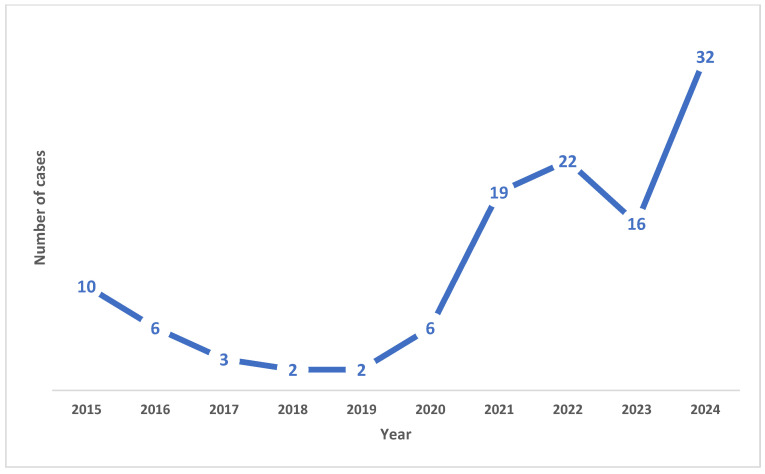
Trends of animal poisoning reported to Ramathibodi Poison Center.

A total of 118 animal poisoning cases recorded by Ramathibodi Poison Center from 2015 to 2024 were reviewed. The number of consultations on animal poisoning at the center showed a tendency to increase over this period, as shown in Figure 1.

**Figure 2:**
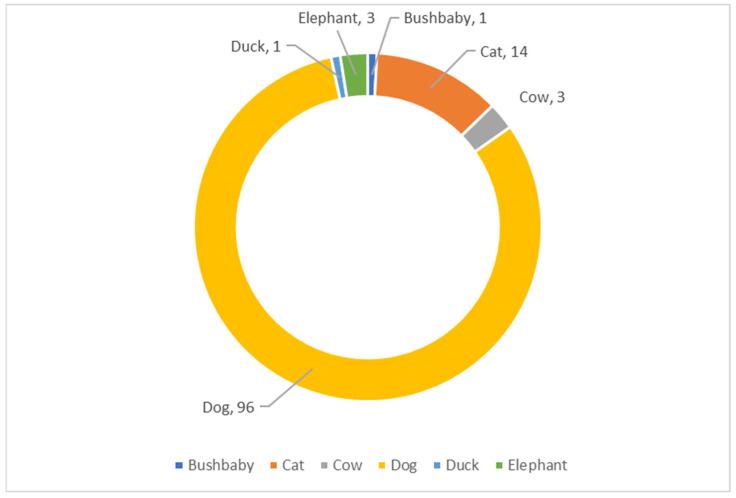
Species distribution of animals involved in poisoning cases reported to Ramathibodi Poison Center.

Most incidents involved companion animals, which accounted for 110 cases (93.2%). Dogs represented the largest affected group, with 96 cases, while cats accounted for 14 cases. The remaining eight cases involved non-pet species, including cows, elephants, ducks, and a bush baby. The species distribution is illustrated in Figure 2.

Neurological signs were the dominant clinical presentation and were recorded in 77 animals, as summarized in Table 1. Alteration of consciousness was the most prevalent neurological finding, with tremors and seizures also commonly reported. Gastrointestinal clinical signs were documented in 43 cases, most of which involved vomiting. Respiratory compromise was less frequent, although hypersalivation and fever were regularly noted as accompanying signs. Management was largely supportive. Intravenous fluid therapy was the intervention most often provided, followed by the administration of activated charcoal. Anticonvulsant treatment, including barbiturates or propofol, was required in a subset of animals, and airway protection with endotracheal intubation was performed when clinically indicated. Specific antidotal therapy, most commonly atropine and snake antivenom, was used in selected cases.

Pesticides such as those for agricultural use were the leading cause of exposure in this cohort, accounting for 49 cases (41.5%). Among other agents, household products were the most frequently implicated, with 18 cases (15.3%). Medical drugs and exposure to poisonous or venomous animals were reported with equal frequency, each contributing to 14 cases. Exposure to plant-derived toxins was documented in 9 animals, and industrial or occupational chemicals in 7 cases. Only a small number of incidents involved other or unidentified toxic substances (Table 2). Most affected animals survived the exposure, but 14 animals (11.9%) died. These fatalities were primarily linked to pesticide exposure, which accounted for nearly half (n = 6) of all deaths. Additional deaths were attributed to plant toxins and industrial or occupational chemicals, with isolated fatal events observed after medical drug exposure, encounters with venomous animals, and exposure to unidentified toxic agents. All fatal cases recorded in this study are summarized in Table 3.

The fatalities associated with pesticide exposure were due to pesticides including herbicides and insecticides. Herbicide-related deaths involved bipyridyl, chloroacetanilide, and phosphinic acid, while insecticide fatalities were attributed to pyrethroid compounds.

Plant-derived toxins were responsible for three deaths. Two of these involved cassava ingestion by elephants, consistent with cyanogenic glycoside toxicity, and one case involved exposure of a cat to an ornamental plant. Moreover, a single fatality was associated with ibuprofen ingestion by a dog. Additional isolated deaths were linked to a neurotoxic snake bite, industrial or occupational chemicals of unknown composition, and an unidentified toxic substance. Clinical factors were also compared between animals that survived and those that died. Neurological and respiratory involvement at presentation was more frequently observed in fatal cases. Respiratory compromise showed a strong association with mortality (*p* = 0.002), and neurological signs were likewise linked to an increased likelihood of death (*p* = 0.016). Gastrointestinal clinical signs approached statistical significance (*p* = 0.050), suggesting a possible trend toward association with poorer outcomes. These associations should be interpreted cautiously, as the small number of fatal cases limits statistical power and the findings should be considered exploratory rather than confirmatory. In contrast, the toxic exposure category, including pesticide involvement, was not associated with a measurable difference in survival (*p* = 0.914) (Table 4).

Two notable cases of cyanide poisoning were reported in elephants following ingestion of cassava roots and leaves. The first case involved a 34-year-old male elephant that consumed a large quantity of wild cassava without its keeper’s monitoring. Approximately 6 h later, the animal developed hypersalivation, generalized weakness, altered consciousness, and tachypnea with accessory abdominal muscle use. Arterial blood gas analysis revealed pH 7.49, pCO_2_ 36.4 mmHg, and HCO_3_^−^ 27.7 mmol/L. Laboratory values were as follows: BUN 6 mg/dL, creatinine 1.5 mg/dL, AST 29 U/L, ALT 17 U/L, ALP 119 U/L, calcium 4.1 mg/dL, and phosphate 4.5 mg/dL. Supportive management with intravenous fluid therapy was initiated. Treatment for cyanide poisoning was started using 25% sodium thiosulfate at a calculated total dose of 500 mg/kg. Based on the elephant’s estimated body weight of 4000 kg, the entire required amount of antidote could not be administered due to its limited availability in the province. Only approximately one-tenth of the calculated dose was given. The elephant died 26 h after ingestion. Whole-blood analysis for cyanide using high-performance liquid chromatography (HPLC) was positive, confirming the diagnosis. The second case involved a 6-year-old male elephant that ingested an excessive amount of cassava roots and leaves in a cassava plantation. Clinical signs developed approximately 12 h after ingestion and included marked weakness and tachypnea. The elephant was found recumbent in a lateral position and was unable to rise or maintain a standing posture. Despite supportive efforts, the animal’s condition progressively deteriorated. Eighteen hours after ingestion, the elephant died. Antidotal therapy could not be administered because the antidote had not yet arrived due to the long time required to transport it to the remote area. Whole-blood analysis for cyanide using high-performance liquid chromatography (HPLC) was positive, confirming cyanide poisoning.

## 4. Discussion

Animal poisoning remains an underrecognized component of toxicological surveillance in many countries, particularly in regions where no centralized veterinary poison registry has been established. The present study provides a decade-long overview of animal poisoning cases reported to a poison center in Thailand and highlights patterns that reflect both environmental exposure risks and human–animal interactions. The predominance of companion animals, particularly dogs, in these Thai cases of poisoning is consistent with reports from poison control databases in North America and Europe, where access to the household and behavioral curiosity increase the likelihood of exposure [1,2,12,13,14,15].

Pesticides represented the leading cause of poisoning in this study. This aligns with observations made in studies from various countries that agricultural and household pesticide availability continues to pose a substantial hazard to animals, especially in regions with active agricultural sectors [16]. Comparable findings have been reported in European surveillance studies and North American poison control databases, where pesticides consistently represent one of the most common causes of animal poisoning. These international observations suggest that pesticide-related exposures remain a global toxicological concern for companion animals [1,2,10,12,13,14,15]. Herbicides such as paraquat and glufosinate remain well-documented causes of severe toxicity and death in both animals and humans [16,17,18]. Glufosinate is a widely used herbicide that inhibits glutamine synthetase, resulting in disruption of nitrogen metabolism and accumulation of ammonia. Experimental and clinical studies have demonstrated that exposure to glufosinate may lead to neurological manifestations, respiratory distress, and gastrointestinal symptoms in animals. Although confirmed cases of glufosinate poisoning in animals are reported less frequently than those involving other herbicides, available evidence indicates that ingestion of glufosinate-containing products can produce clinically significant toxicity. This mechanism-based toxicity provides important context for interpreting poisoning events associated with herbicide exposure and highlights the potential role of glufosinate among toxic agents affecting animals [18,19]. The recording of a case of fatal pyrethroid exposure further underscores that compounds often perceived as having low toxicity may still produce life-threatening outcomes when exposure is significant or treatment is delayed [20]. These results emphasize the need for improved pesticide stewardship and public education regarding safe storage. Although pesticides accounted for a large proportion of fatal narratives, statistical comparison did not demonstrate a measurable difference in survival between pesticide and non-pesticide exposures. This discrepancy likely reflects the limited sample size and highlights the distinction between frequency of exposure and quantified mortality risk.

Neurological manifestations dominated the clinical presentations in this study, a pattern frequently observed in toxic exposures involving pesticides, plant toxins, and neuroactive pharmaceuticals [21]. The strong association between respiratory compromise and mortality observed in our study is clinically plausible, as respiratory failure often represents the terminal pathway in severe poisoning events [22]. Similarly, neurological depression may indicate systemic toxicity and impending physiological collapse. These associations have been described in both veterinary emergency settings and the literature on human toxicology, supporting the translational relevance of poison center surveillance [11,23].

Although most of the animals included in this study survived, the mortality rate of nearly 12% is not trivial. Fatalities were concentrated in cases of exposure involving highly toxic agents such as paraquat and cyanogenic plant toxins. Meanwhile, the cassava-associated cyanide toxicity in elephants illustrates how regional dietary or environmental factors influence species-specific risk [24]. Such findings highlight the importance of geographically contextual toxicology data, as exposure patterns vary significantly between countries and ecosystems.

Management strategies in this cohort were largely supportive, reflecting current best practice in veterinary toxicology, where early stabilization and symptomatic care remain the cornerstone of treatment [9,14,25]. When available, atropine and antivenom appear to be effective for cases of pesticides or snake envenomation. However, the variability in available treatments across regions of Thailand reinforces the need for poison center consultation networks that support frontline clinicians.

Although rare, these elephant cases illustrate region-specific toxic hazards that may not appear in conventional veterinary registries but are highly relevant in agricultural ecosystems where humans and large herbivores share exposure pathways. Raw cassava contains harmful substances, most notably cyanogenic glycosides, which release hydrocyanic acid upon hydrolysis [26,27]. This compound is highly toxic and has been documented to cause poisoning in multiple animal species, including cattle, sheep, pigs, and poultry [28,29]. Despite the well-established toxic potential of cassava in veterinary toxicology, to the best of our knowledge, cyanide poisoning associated with cassava ingestion has not previously been reported in elephants. These findings expand the current understanding of cassava toxicity and highlight a previously unrecognized risk in large herbivores exposed to cyanogenic plants.

This study also demonstrates the value of centralized poison surveillance systems covering cases in both clinical and veterinary medicine. Ramathibodi Poison Center functions as a national toxicological reference point, and its registry captures trends that may otherwise remain undocumented. Poison center databases have repeatedly been shown to serve as early warning systems for emerging toxic threats in both animals and humans. In several countries, national poison center networks have been successfully used to detect emerging toxic exposures, monitor poisoning trends, and guide preventive public health interventions. These systems demonstrate how centralized toxicological surveillance can contribute to both veterinary and human health protection [30,31]. Strengthening such surveillance infrastructure has implications not only for veterinary care but also for public health preparedness. Animal poisoning events extend beyond veterinary concern and function as environmental sentinels of chemical risk within shared human–animal ecosystems. Patterns observed in companion animals may reflect unsafe pesticide storage, household chemical exposure, and agricultural practices that simultaneously threaten human health. Integrating veterinary poison surveillance into broader public health monitoring frameworks may therefore enhance early detection of hazardous exposures and support preventive policy development within a One Health model [32].

This study has limitations inherent to retrospective poison center research. Reporting bias likely favors moderate-to-severe cases, while mild exposures may go undocumented. In addition, follow-up outcomes depended on available clinical information, and the sample reflects consultation behavior rather than true population incidence. Consequently, the findings should be interpreted as consultation-based surveillance data rather than true national incidence estimates. Nevertheless, poison center datasets remain among the most powerful tools for identifying real-world exposure patterns, particularly in regions lacking formal veterinary reporting systems.

Taken together, the findings of this study reinforce the assertion that animal poisoning is a multifactorial issue situated at the intersection of environmental safety, human behavior, and veterinary medicine. Continued surveillance, targeted prevention strategies, and improvements in public awareness via programs such as information campaigns are essential to reduce avoidable exposures. Future work should integrate veterinary and human toxicology databases within a One Health framework to better characterize shared environmental risks.

## 5. Conclusions

This 10-year surveillance study suggests that animal poisoning in Thailand is primarily driven by pesticide exposure and frequently presents with neurological compromise, with a non-negligible risk of preventable death. Companion animals, particularly dogs, remain the most vulnerable population. Respiratory and neurological involvements at presentation were strongly associated with mortality and should thus be urgently prioritized in clinical settings. These findings highlight the value of centralized poison surveillance and emphasize the need for improved pesticide stewardship and integrated veterinary public health monitoring to reduce avoidable toxic exposures.

## Figures and Tables

**Table 1:** Clinical signs and treatments administered in animal poisoning cases.

Clinical Signs and Treatment		Number (N = 118)
**Clinical signs**	**Gastrointestinal clinical signs**	
	Vomiting	38 (32.2%)
	Retching/gagging	5 (4.2%)
	**Neurological clinical signs**	
	Alteration of consciousness	35 (29.7%)
	Tremor	16 (13.6%)
	Seizure	13 (11.0%)
	Weakness	13 (11.0%)
	**Respiratory clinical signs**	
	Dyspnea	10 (8.5%)
	Tachypnea	7 (5.9%)
	**Other**	
	Fever	7 (5.9%)
	Hypersalivation	29 (24.6%)
**Management**	**Supportive Treatment**	
	Intravenous fluid	48 (40.7%)
	Activated charcoal	21 (17.8%)
	Anticonvulsants	12 (10.2%)
	Endotracheal intubation	11 (9.3%)
	Antiemetic drug	4 (3.4%)
	Antibiotics	4 (3.4%)
	**Specific Treatment**	
	Snake antivenom	10 (8.5%)
	Atropine Cyanide antidote	13 (11.0%) 1 (0.8%)

**Table 2:** Outcomes of animal poisoning cases stratified by exposure category.

Group of Categories		Survive	Death
Pesticides		43 (87.8%)	6 (12.2%)
Non-Pesticides	Household Products	18 (100%)	0
	Medical Drugs	13 (92.9%)	1 (7.1%)
	Poisonous and Venomous Animals	13 (92.9%)	1 (7.1%)
	Plant Toxins/Poisonous Plants	6 (66.7%)	3 (33.3%)
	Technical and Occupational Products	5 (71.4%)	2 (28.6%)
	Other Category	5 (100%)	0
	Unknown Toxic Substance	1 (50.0%)	1 (50.0%)

**Table 3:** Characteristics of fatal animal poisoning cases.

Category of Death Cases		Number/Animal
**Medical Drugs**		
Ibuprofen		1/Dog
**Pesticides**		
**Herbicides**		
Bipyridyl		1/Dog
Chloroacetanilide		1/Cow
Phosphinic acid		1/Dog
**Insecticides**		
Pyrethroid		3/Dogs
**Plant Toxins/Poisonous Plants**		
Cyanogenic glycoside		2/Elephants
*Zamioculcas zamiifolia*		1/Cat
**Poisonous and Venomous Animals**		
**Poisonous Snake: Neurotoxin**		
Cobra snake		1/Dog
**Technical and Occupational Products**		
Unknown Technical & Industrial chemicals		2/Dogs
**Unknown Toxic Substances**		
Unknown Toxic Substance		1/Dog

**Table 4:** Factors associated with mortality in animal poisoning cases.

Factor	Death, n (%)	Survival, n (%)	*p*-Value
Species (Pet/Non-pet)			
Pet	11 (78.6%)	99 (95.2%)	0.053
Non-pet	3 (21.4%)	5 (4.8%)	
Category			
Pesticides	6 (42.9%)	43 (41.5%)	0.914
Non-Pesticides	8 (57.1%)	69 (58.5%)	
Clinical signs (initial)			
Gastrointestinal clinical signs	8 (57.1%)	32 (30.8%)	0.050
Neurological clinical signs	9 (64.3%)	31 (29.8%)	0.016
Respiratory clinical signs	6 (42.9%)	8 (7.7%)	0.002
Other	7 (50.0%)	38 (36.5%)	0.330
Treatment			
Specific treatment	5 (35.7%)	17 (16.4%)	0.135
Symptomatic treatment	9 (64.3%)	87 (83.6%)	

## Data Availability

The original contributions presented in this study are included in the article. Further inquiries can be directed to the corresponding author.
